# Supplementary figures and images for: How accurate is circular dichroism-based model validation?
Source: Eur Biophys J. 2020 Aug 26;49(6):497–510. doi: 10.1007/s00249-020-01457-6 (PMC7456416; doi:10.1007/s00249-020-01457-6)

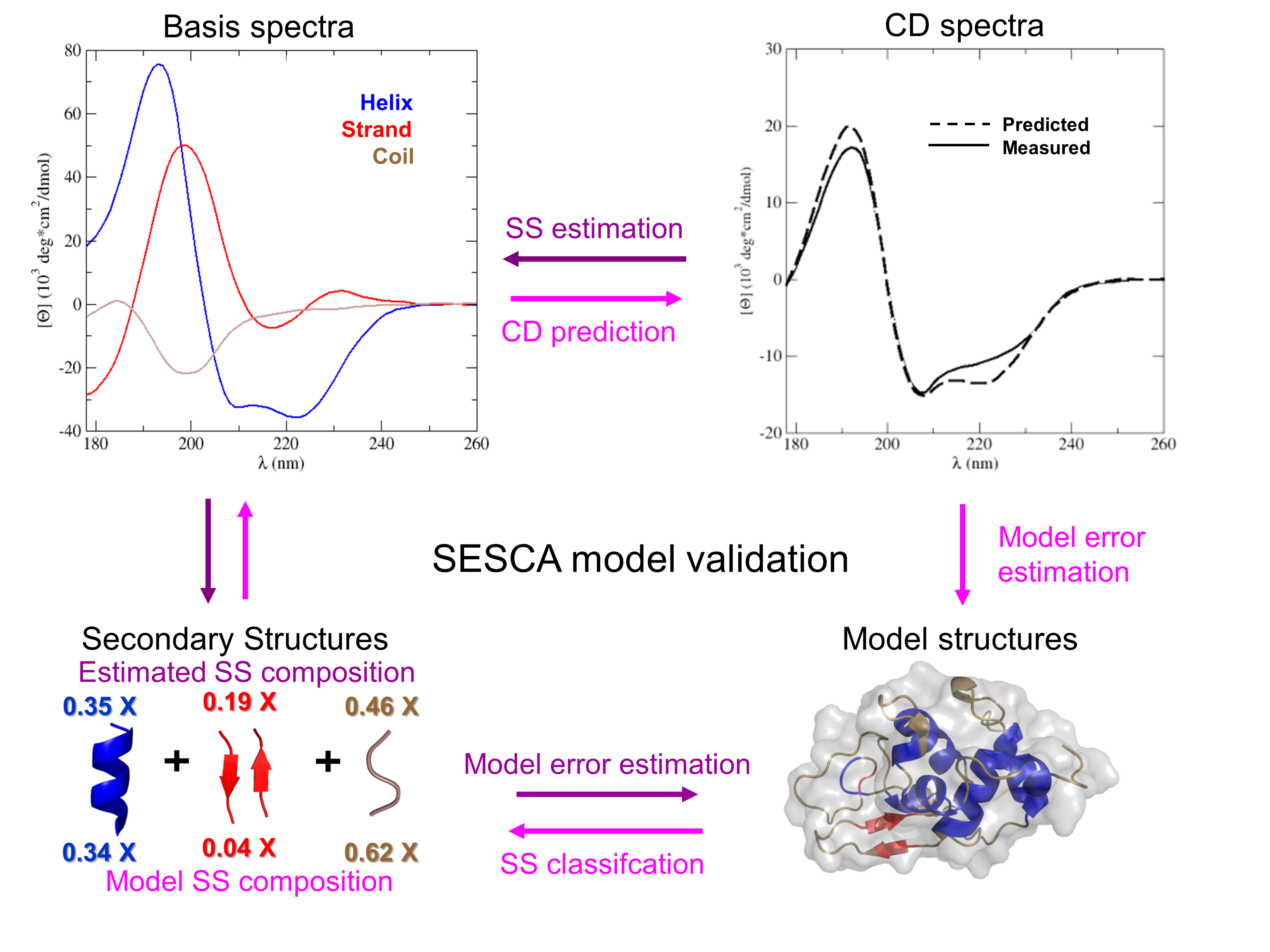

Supplement: Supplementary file 1 — Figure S1: Model validation schemes. In SESCA, the model quality is assessed based on either CD spectrum predictions or SS estimations. In the former case, the SS composition of structural models is extracted by a classification algorithm, and used as weighting factors for the basis spectra of SS elements to predict the CD spectrum. The quality of a model is estimated based on the deviation of its predicted CD signal from measured CD spectrum. In the latter case, the measured CD spectrum is approximated by a linear combination of the basis spectra to yield an estimated SS composition, and model quality is assessed based on the deviation of model SS compositon from the obtained SS estimate. [file 249_2020_1457_MOESM1_ESM.png]
